# Brain Computer Interface Systems for Neurorobotics: Methods and Applications

**DOI:** 10.1155/2017/2505493

**Published:** 2017-10-30

**Authors:** Victor Hugo C. de Albuquerque, Robertas Damaševičius, Nuno M. Garcia, Plácido Rogério Pinheiro, Pedro P. Rebouças Filho

**Affiliations:** ^1^Programa de Pós Graduação em Informática Aplicada, Laboratório de Bioinformática, Universidade de Fortaleza, Fortaleza, CE, Brazil; ^2^Department of Software Engineering, Kaunas University of Technology, Kaunas, Lithuania; ^3^Department of Informatics, Instituto de Telecomunicações and ALLab Assisted Living Computing and Telecommunications Laboratory, Universidade da Beira Interior, Covilhã, Portugal; ^4^Laboratório de Processamento de Imagens e Simulação Computacional, Instituto Federal de Educação, Ciência e Tecnologia do Ceará, Maracanaú, CE, Brazil

Brain computer interface (BCI) systems establish a direct communication between the brain and an external device. These systems can be used for entertainment, to improve the quality of life of patients and to control virtual and augmented reality applications, industrial machines, and robots. In the neuroscience field such as in neurorehabilitation, BCIs are integrated into controlled virtual environments used for the treatment of disability or cognitive development of subjects, for example, in case of cerebral palsy, Down syndrome, and depression. Its aim is to promote a recovery of brain function lost due to a lesion through noninvasive brain stimulation (brain modulation) in a more accurate and faster manner than the traditional techniques. Neurorobotics combines BCIs with robotics aiming to develop artificial limbs, which can act as real members of human body being controlled from a brain-machine interface. With the advancement of a better understanding of how our brain works, new realistic computational algorithms are being considered, making it possible to simulate and model specific brain functions for the development of new Computational Intelligence algorithms and, finally, BCI for mobile devices/apps.

As an augmentative communication channel, BCI has already attracted considerable research interest thanks to recent advances in neurosciences, wearable biosensors, and data mining. However, to overcome numerous challenges the BCI technology still requires research in high-performance and robust signal processing and machine learning algorithms to produce a reliable and stable control signal from nonstationary brain signals to allow development of real-life BCI systems usable across many individuals. Further improvements to BCI systems are necessary to ensure that they can meet the needs of specific user groups such as disabled or impaired people as well as common users.

The main objective of this special issue is to promote a discussion on the recent advances related to BCI systems for neurorobotics from novel methods and/or applications in order to identify innovative, current, and great contribution works to the field of neuroscience. This special issue contains 09 published original works selected from 13 submitted articles, addressing new trends in the area from several novel methods and techniques used in different applications. For example, Q. Gao et al. presented a novel hybrid BCI using EEG signal, which consists of a motor imagery-based online interactive brain-controlled switch, aka “Teeth Clenching” state detector, and a SSVEP-based BCI was proposed to provide multidimensional BCI control. N. Yu et al. proposed a novel method for extracting the single-trial evoked potential (EP) based on multiple-input single-output autoregressive modeling with exogenous input (MISO-ARX). F. A. Araújo et al. proposed the Auris System, based on a noninvasive brain activity recording using a electroencephalographic device, conceived to provide the musical experimentation for people who have some type of hearing loss, being able to extract musical information from audio and create a representation for music using different stimuli, a new media format to be interpreted by other senses than the hearing. A. M. Batula et al. used, for the first time, four-class motor imagery-based online functional near infrared spectroscopy BCIs adopted to control a robot with upper- and lower-limb movement imagery mapped to four high-level commands to control the navigation of a simulated or real robot in a room. A significant improvement in classification accuracy was found between the control of virtual robot-based BCI and the real robot BCI. L. Carelli et al. accomplished the systematic review of BCI for cognitive assessment and training, describing some preliminary attempts to develop verbal-motor free BCI-based tests for evaluating specific or multiple cognitive domains in patients with Amyotrophic Lateral Sclerosis (ALS), disorders of consciousness, and other neurological diseases, presenting the more heterogeneous and advanced field of BCI-based cognitive training, which has its roots in the context of neurofeedback therapy and addresses patients with autism spectrum disorder (ASD) and attention deficit hyperactivity disorder, stroke patients, and elderly subjects. Z. Lin et al. proposed a triple-rapid serial visual presentation (RSVP) paradigm with three types of image retrieval simultaneously and a target image appearing three times to improve the detection accuracy of the multitrial P300-based classification methods. C. A. D. R. Paula et al.'s research aims to evidence quantitative differences in the frequency spectrum pattern between EEG signals of children with and without ASD during analysis of human facial expression, such as neutral, happy, and angry. A. Athanasiou et al. proposed an off-the-shelf BCI-controlled anthropomorphic robotic arms involving, mainly, social Human-Robot Interaction for assistive technologies and rehabilitation. N. Zhuang et al. perform emotion recognition based on the empirical mode decomposition (EMD) of EEG signals.

